# Modelling pool testing for SARS-CoV-2: addressing heterogeneity in populations

**DOI:** 10.1017/S0950268820003052

**Published:** 2020-12-28

**Authors:** Javier Fernández-Salinas, Diego Aragón-Caqueo, Gonzalo Valdés, David Laroze

**Affiliations:** 1 Escuela de Medicina, Universidad de Valparaíso, Valparaíso, Chile; 2 Departamento de Ingeniería Industrial y de Sistemas, Universidad de Tarapacá, Casilla 7D, Arica, Chile; 3 Instituto de Alta Investigación, CEDENNA, Universidad de Tarapacá, Casilla 7D, Arica, Chile

**Keywords:** Coronavirus, modelling, pool testing, public health, strategy

## Abstract

Amplifying the testing capacity and making better use of testing resources is a crucial measure when fighting any pandemic. A pooled testing strategy for SARS-CoV-2 has theoretically been shown to increase the testing capacity of a country, especially when applied in low prevalence settings. Experimental studies have shown that the sensitivity of reverse transcription-polymerase chain reaction is not affected when implemented in small groups. Previous models estimated the optimum group size as a function of the historical prevalence; however, this implies a homogeneous distribution of the disease within the population. This study aimed to explore whether separating individuals by age groups when pooling samples results in any further savings on test kits or affects the optimum group size estimation compared to Dorfman's pooling, based on historical prevalence. For this evaluation, age groups of interest were defined as 0–19 years, 20–59 years and over 60 years old. Generalisation of Dorfman's pooling was performed by adding statistical weight to the age groups based on the number of confirmed cases and tests performed in the segment. The findings showed that when the pooling samples are based on age groups, there is a decrease in the number of tests per subject needed to diagnose one subject. Although this decrease is minuscule, it might account for considerable savings when applied on a large scale. In addition, the savings are considerably higher in settings where there is a high standard deviation among the positivity rate of the age segments of the general population.

## Introduction

Testing for early recognition of infection sources and cutting off transmission forms the cornerstone of any public health response to emerging outbreaks [1]. Increasing a country's testing capacity to identify infected individuals and to contain the spread of the virus is a crucial strategy [1, 2]. Most countries have been ramping up their testing capacity to different degrees; some are doubling it in a matter of weeks, while others are steadily increasing their capacity in a more linear pattern [3]. However, amplifying the testing capacity is still an ongoing task to face the pandemic. To improve the use of limited resources and to obtain the most out of each testing kit, implementing a pool testing strategy has been proposed [4].

The foundational work for this strategy dates back to 1943, based on Dorfman's pooling, who first introduced the concept of pooling clinical specimens to save on testing resources [5]. This strategy could potentially increase worldwide testing capacity many times over [6], if used correctly, in the right segment of the population and under the specific historical prevalence of positive results [7, 8] and test sensitivity [9]. It has also shown promising results as a screening tool in clinical practice [10, 11], and it has been implemented on a large scale to test asymptomatic individuals, showing a considerably increased throughput in testing coverage while maintaining test sensitivity [12]. In addition, the WHO has recently established that group testing for SARS-CoV-2 is a feasible strategy that can increase testing capacity and can be applied in low prevalence settings. However, it does not recommend routine pooling in laboratories or using pooled samples for contact tracing purposes [13].

Several pooling strategies have been proposed [14, 15]. One that adapts to the clinical reality and could potentially be implemented in the healthcare setting establishes that multiple samples are grouped in a pooled sample, and a single reverse transcription-polymerase chain reaction kit is used to test that unified sample. If the test comes out negative, then the infection is ruled out in all of the individuals included in the pooled sample. If the test comes out positive, then all of the individuals in that group have to be retested individually [5, 6]. This strategy has been previously implemented to test for other pathogens before an outbreak. Examples include HIV [16, 17], chlamydia [18], influenza [19], cytomegalovirus [20] and many others, concluding that it is a cost-saving strategy that increases overall testing capacity in a clinical context where the prevalence of the pathogen is low. Since the peak of the pandemic has already passed in most countries [21], positivity rates remain relatively low. This scenario makes it especially suitable for the pool testing strategy to be implemented since, in the context of a low prevalence, this approach is more efficient.

Regarding SARS-CoV-2, experimental studies suggest that pooling nasopharyngeal samples under a 10% prevalence of positives translates into considerable savings [22], with no decrease in sensitivity in groups of five samples [6]. Additionally, other studies have shown that a single positive can be detected in groups of up to 32 samples [23]; however, there is a 10% margin of error. Moreover, the viral load might play a decisive role in the sensitivity of the pooled samples [24], and targeting two genes of SARS-CoV-2 might increase the efficiency of the detection of positive samples in minipools of 5–10 samples [25]. However, since the pooling of specimens might reduce the test sensitivity due to pooling dilution, the optimal group sizes should be thoughtfully estimated [26, 27]. In addition, it is crucial to establish guidelines for efficient and accurate pooling algorithms that will ensure maximum throughput of the strategy when implemented in the clinical context [28].

Previous models for estimating the optimum group size based on the historic positive prevalence have been proposed [5, 7, 9, 27]. Nevertheless, since the calculations are based on historical prevalence, they assume a homogeneous distribution of the population contained in that prevalence, and it is clear based on the clinical context that this does not hold true. Taking into account the distribution of confirmed cases among age groups, it can be observed that some countries have the majority of their confirmed cases in the young adult population, while other countries have it among the elderly population [29]. Therefore, it becomes prudent to explore how the particular distribution of confirmed cases among age groups might play a role in the problem of pooling optimisation.

The aim of this study is to explore whether there is any improvement in test savings based on Dorfman's probabilistic model for estimating the group size when individuals are separated by age groups when grouped into equally sized pooled samples among the age categories.

## Materials and methods

The groundwork for this generalisation is Dorfman's pooling [5], which has been applied for SARS-CoV-2 [7]. In this model, the estimation of optimum group size (*n*) for the implementation of pool testing was performed based on the historical prevalence of positives (*x*) as the input. This model estimated group sizes that ranged from 11 to 3 subjects, for a prevalence of positives from 1% to 30%. It predicted a 40.6% saving of tests for a prevalence of 10%, using groups of 4, and a 17.9% saving for a prevalence of 20% with groups of 3. The model flattens when the prevalence reaches or exceeds 30%, and the strategy is no longer useful compared to individual testing. The main findings provide a relationship for the number of tests per subject needed to diagnose one subject (*z*) using a pool testing strategy, which is denoted by *z* = *z*(*n*, *x*), where *n* stands for the optimum group size and *x* stands for the historical prevalence of positive tests in a particular context. In particular, *z* can be further derived in the following form:1
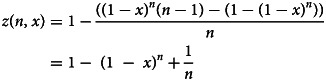
We remark that *n* can be estimated using the global minimum at a given *x*. However, as mentioned above, using *x* as an input assumes a homogeneous distribution of the infected individuals, which differs from the clinical reality. Therefore, adding statistical weight to different epidemiological features known to date and addressing how those features may fit the individual who is being included in the pooled sample further optimises the estimation of *n*. For this particular case, we will explore how age groups might affect the *n* and *z* that the model predicts. Knowing that *x* is a function of many factors that are included together to represent an apparent homogeneous distribution, to isolate how pooling by age might affect the overall performance of the pool testing strategy, a statistical weight is added to the age segments of interest. This weight is estimated based on the portion of testing that each age group receives, which is defined as an empirical parameter. The reason for this is because this generalisation assumes that the distribution of testing among the age groups is not proportional to the age distribution of the general population under study, thus implying that some groups receive a greater proportion of the available tests than others.

Let us remark that this model is developed so that the prevalence of positives in each age segment is proportional to the overall prevalence of positives; thus, the generalisation is governed by the global prevalence of positives rather than the particular prevalence in each segment of interest. In this way, the pool sizes estimated are the same throughout the segments.

Note that the efficiency of pooling and group sizes predicted by this model is compared here to two other models. First, we used standard Dorfman's pooling and second, we used Dorfman's pooling separated by age groups, using the prevalence of each age segment individually.

### Generalisation: addressing heterogeneity

In this case, we consider that there are different population segments to account for. That is, we now consider that the generalised function of Eq. (1) becomes a linear combination of *z* functions weighted by constants, ɛ_*i*_, where *i* = 1, …, *N*, such that *N* represents the number of segments. This generalisation is intended to capture the heterogeneity between the population segments. This heterogeneity arises from the different positivity rates among the age segments, unequally represented in the total tests. Therefore, different segments show up weighing differently in the optimisation problem. The involved friction captured by the heterogeneity parameter, ɛ_*i*_, could be related to age groups, demographic segregation, sampling time mismatch and many others, but for this case, it will be related to age groups.

Therefore, it is necessary to account for this heterogeneity in *x* and remove the assumption from the basic model in which a homogeneous distribution across segments was assumed. Then, let us consider that for each segment, the prevalence is also weighted by factors *γ*_*i*_. Therefore, the new objective function, *z*_new_, represents the generalisation idea explained before and can be explicitly written as:2
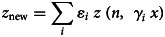
This means there is a distribution across segments characterised by *x*_*i*_ = *γ*_*i*_*x* such that the whole population's historical prevalence of positive tests is *x*. From this, the prevalence of positives in a segment can be defined as a function of the global prevalence, thus facilitating the generation and comparison of various heterogeneous scenarios with different global positivity but, eventually, with a proportional distribution of cases. In other words, this generalisation implies that there is a population parameter *x* that is the resulting linear combination of the segment's specific parameters *x*_*i*_. Since we know the statistical measure for the whole population's historical prevalence of positive tests, *x*, we construct a distribution around that known value to characterise the variability of prevalence among the groups. Certainly, it is possible to define this distribution in other ways as well. From these, ɛ_*i*_ is determined as the test performed in the segment, divided by the total tests performed, while *γ*_*i*_, as mentioned before, is defined as the prevalence in the segment (*x*_*i*_) divided by the global prevalence (*x*).

Next, let us analyse the case of *N* = 3. For this purpose, we assume that three groups of interest are established, which will later be defined as age groups from 0 to 19, 20 to 59 and >60. To provide closure of the linear superposition, we can assume that the total statistical weight should be one; this implies that 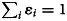. On the contrary, the total prevalence remains a global parameter 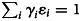. Therefore, at *N* = 3, when (*ɛ*_*i*_, *γ*_*i*_) are established for the first two groups of interest, the third closing group should meet the following conditions: *ɛ*_3_ = 1 − *ɛ*_1_ − *ɛ*_2_, *γ*_3_ = (1 − *γ*_1_*ɛ*_1_ − *γ*_2_ *ɛ*_2_)/(1 − *ɛ*_1_ − *ɛ*_2_), such that *ɛ*_1_ + *ɛ*_2_ ≠ 1. To quantify heterogeneity in the possible combinations of the parameters, the standard deviation (*σ*) of the prevalence of positives can be calculated as follows:3
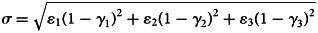
From Eq. (2), two numerical experiments appear to be interesting. The first is related to evaluating *z* and *n* for different country scenarios, as each country will have a particular combination of *ɛ*_*i*_ and *γ*_*i*_ for each age segment. The second considers assessing whether there is a formula describing the variation of *z* as a function of heterogeneity *σ*.

### Determining empirical values for *ɛ* and *γ*

As mentioned before, when generalising Dorfman's pooling for evaluating how pooling by age groups might affect the overall performance of the strategy, three age groups of interest arise. The first segment will be defined as individuals between the ages of 0 and 19 years old who, due to the closure of schools, are likely to have stayed at home. The second group will be defined as *working-class* adults, ranging from 20 to 59 years of age. Finally, and represented by the closing function, the third group of interest will be defined as the older adult population of 60 years old and above. This is because they are probably no longer part of the working population and are also likely to stay at home.

Unfortunately, a testing distribution by age group data is not available for most countries. Most epidemiological reports usually report the total number of tests performed and the number of confirmed cases. The confirmed cases are then further classified into age groups. However, out of all of the tests performed, there is no report on the age groups among the population getting tested.

Nevertheless, publicly available information published by the Australian state of New South Wales (NSW) [30] and information obtained via the Transparency Law from two Chilean hospitals, Hospital Calvo Mackenna, Santiago and Hospital Grant Benavente, Concepción, were used as references. Let us remark that the samples reported by the Chilean hospitals included in this study group the processing samples of both the hospital itself and all of the primary care centres across the territory that the hospital covers. The NSW state reported between the 9th of March and the 24th of April 2020 a total of 193 716 tests, from which 2943 were positive, yielding an overall prevalence of positives of 1.5%. On the contrary, the Hospital Calvo Mackenna reported 14 586 tests performed up to the 16th of July, with 5730 being positive, yielding an overall positivity rate of 39.3%. Finally, the Hospital Grant Benavente reported up to the 26th of July 35 068 tests performed, of which 5205 were positive, yielding an overall prevalence of positives of 14.8%. From these, the estimated *ɛ* and *γ* values are summarised in Table 1.

**Table 1.** Different *ɛ* and *γ* values obtained in specific scenarios

	New South Wales state (AUS) (*n* = 193 716)	H. Calvo Mackenna (CHL) (*n* = 14 586)	H. Grant Benavente (CHL) (*n* = 35 068)
*ɛ*_1_	0.101	0.196	0.129
*ɛ*_2_	0.691	0.626	0.692
*ɛ*_3_	0.208	0.178	0.179
*γ*_1_	0.396	0.582	0.789
*γ*_2_	0.941	1.082	1.090
*γ*_3_	1.489	1.171	0.804

It is important to add that *γ*_3_ and *ɛ*_3_ arise from the closing function but are shown in the table for a better comprehension of the scenarios described.

### Generalisation of z according to heterogeneity (σ)

To evaluate whether there is a determined relationship between the optimal *z* as a function of heterogeneity (*σ*) and between the percentage decrease of the optimal *z* with respect to the *z* obtained in the initial model (1) (where *σ* is assumed to be 0), a scatter plot of (1) as a function of *σ* for six different prevalences (5%, 10%, 15%, 20%, 25% and 30%) will be presented. In case a trend is observed, the function of best fit describing this trend will be calculated through nonlinear regression.

The data composing these scatter plots will correspond to 149 pairs of *z* and *σ* for each of the six analysed prevalences (*x*) that arise from solving Eq. (2) for five different combinations of *ɛ*_1_ and *ɛ*_2_ (0.1–0.2, 0.1–0.3, 0.2–0.6, 0.3–0.4, 0.3–0.5) and 30 *γ* combinations (*γ*_1_ varying from 0.4 to 0.8, and *γ*_2_ varying from 0.9 to 1.4, both with intervals of 0.1). Finally, *ɛ*_3_ and *γ*_3_ will be determined in each case by their closure function. It is important to mention that a combination was discarded since it presented a negative *γ*_3_, which is not possible in reality.

### Generalisation: addressing specific scenarios

Having introduced all of the parameters that will come into play when estimating *n* when the pool groups are separated by age, specific simulations based on the state and hospital data can be established. Since *γ*_2_ accounts for the age segment of individuals from 20 to 59, with the highest number of confirmed cases worldwide [25], it will vary from 0.8 to 1.2 in the intervals of 0.2 because, as seen in Table 1, this is the range of possible values that *γ*_2_ might take.

Finally, let us comment that all of the computations were performed with software Wolfram Mathematica, along with Microsoft Excel 365 for the nonlinear regressions [31, 32].

### Ethical aspects

It is important to mention that no approval from an Ethics Committee is necessary since the information from the NSW is publicly available on its website, while the data from Chile are subject to the Transparency Law, where each institution providing the information guarantees that the data provided do not compromise/detract the anonymity of the patients and is also considered information in the public domain.

## Results

### *z* in particular countries

**Fig. 1.**
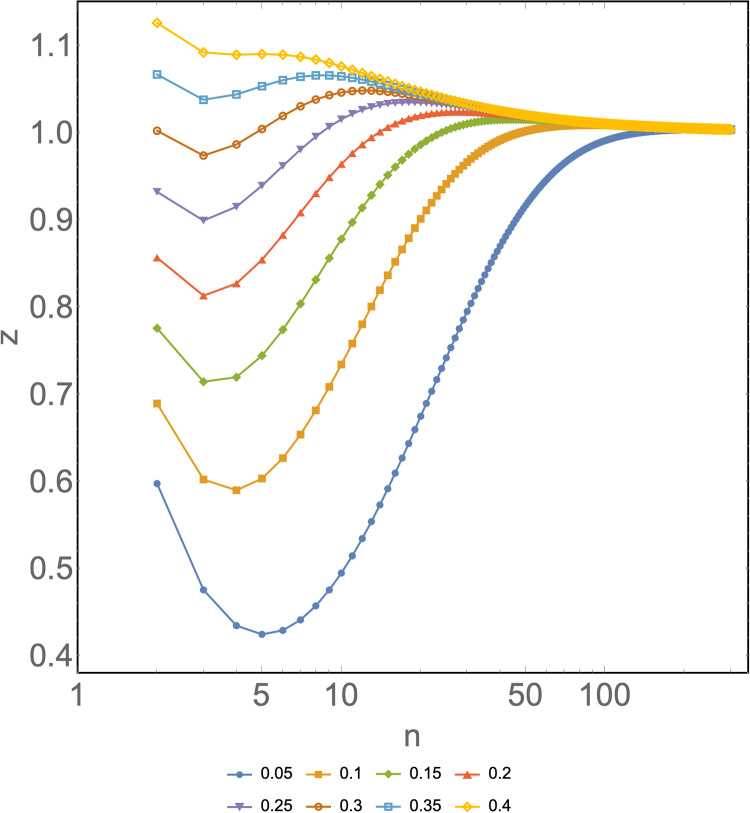
*z* as a function of *n* for different prevalences (*x*) ranging from 0.05 to 0.4 for the particular case of New South Wales state, Australia. Horizontal axis: group size of a pooled sample (*n*). Vertical axis: number of tests per subject needed to diagnose one subject (*z*). Different colours represent different prevalences. Input: *ɛ*_1_ = 0.101, *ɛ*_2_ = 0.691, *ɛ*_3_ = 0.208, *γ*_1_ = 0.396, *γ*_2_ = 0.941 and *γ*_3_ = 1.489.

Optimal values were observed in scenarios of a low prevalence of positives, ranging from 0.05 to 0.3 regardless of the country. The global minimum indicates where the optimal value of *z* is as a function of *n* at a given *x*. Figure 1 shows the local minima obtained in the model adapted to Australia, specifically for the New South Wales state, since it had the most complete and available data and thus serves as the scenario in which the model better adapts to a specific reality.

Chile and Australia (as shown in Fig. 1) showed their last local minimum at a prevalence of 0.3. From then on, the model becomes undefined, the curve tends to flatten or fall, and there is no global minimum. As mentioned earlier, *ɛ* stands for the specific statistical weight for a given age group. These remain reasonably fixed or change little over time. However, since *γ* represents the relative risk for a particular group and is heavily influenced by the newly confirmed cases that come up every day, this parameter is rather dynamic; thus, it needs to be varied across possible values that it could take as the pandemic progresses.

Table 2 summarises the main results that arise from varying *γ*_2_ across the segments from 0.8 to 1.2, which are the possible values that *γ*_2_ might take at different historical prevalences based on the specific context of each country included in the simulations.

**Table 2.** Summary of the main findings for *n* and *z* from varying *γ*_2_ across different scenarios

*γ*_2_	*x*	New South Wales (AUS)		H. Calvo Mackenna (CHL)		H. Grant Benavente (CHL)	
		*n*	*z*	*n*	*z*	*n*	*z*
0.8	0.05	5	0.421	5	0.42	5	0.422
	0.1	4	0.582	4	0.58	4	0.585
	0.15	4	0.705	4	0.702	3	0.709
	0.2	3	0.798	3	0.795	3	0.804
	0.25	3	0.878	3	0.873	3	0.887
	0.3	3	0.946	3	0.941	3	0.959
1.0	0.05	5	0.425	5	0.425	5	0.426
	0.1	4	0.591	4	0.59	4	0.593
	0.15	3	0.716	3	0.715	3	0.719
	0.2	3	0.816	3	0.814	3	0.82
	0.25	3	0.903	3	0.901	3	0.91
	0.3	3	0.98	3	0.977	3	0.988
1.2	0.05	5	0.424	5	0.425	5	0.424
	0.1	4	0.589	4	0.59	4	0.589
	0.15	4	0.714	3	0.715	3	0.713
	0.2	3	0.812	3	0.814	3	0.811
	0.25	3	0.898	3	0.901	3	0.896
	0.3	3	0.972	3	0.977	3	0.97

### Generalisation of z values in the function of σ

**Fig. 2.**
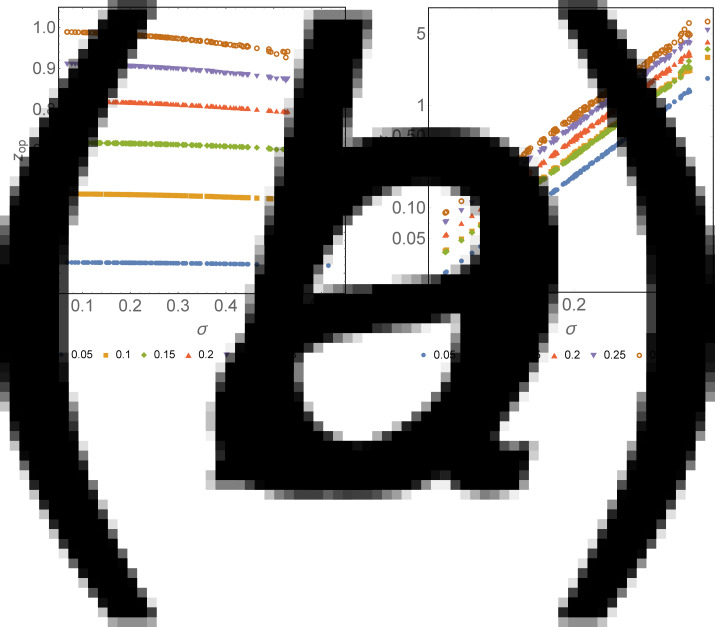
(a) Optimum *z* as a function of *σ* for different prevalences colour-coded, from 0.05 to 0.3. Horizontal axis: heterogeneity of the population (*σ*). Vertical axis: optimum number of tests needed to diagnose one subject (*z*) based on the optimum group size previously calculated. (b) Relative percentage decrease of the optimum *z* as a function of *σ* with respect to the optimum *z* estimated for *σ* = 0 expressed in logarithmic form. Horizontal axis: heterogeneity of the population (*σ*). Vertical axis: percentage decrease of the optimum *z* as a function of *σ* with respect to the optimum *z* estimated for *σ* = 0.

On the contrary, as shown in Figure 2a, it can be observed that for all values of *x*, the optimum value of *z* decreases as the heterogeneity of the tested population increases (*σ*). However, this decrease is not equal for all cases, showing a more significant reduction at the higher prevalence spectrum. For a 30% prevalence of positives, there is a net decrease of 0.065 in *Z*_op_ when it goes from *z*_*σ*=0_ = 0.99 to *z*_*σ*=0.614_ = 0.925. On the contrary, for a low prevalence, the case that varies the least is for a 5% positivity, showing a decrease of 0.008 in the *Z*_op_ from *z*_*σ*=0_ = 0.426 to *z*_*σ*=0.614_ = 0.418. This translates into an additional benefit compared to the classic pool testing strategy, where *σ* is assumed to be 0. This benefit ranges from 1 extra test every 14.1 tests for a 30% prevalence to 1 extra test every 22.2 tests for a 5% prevalence at the highest spectrum of *σ*.

The optimal value of *z* can be obtained as a function of *σ* for different prevalences. It can be expressed through the following second-degree polynomial regressions:4
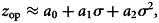
where the constant {*a_j_*} is a function of the prevalence. In Figure 2b, the percentage decrease of the optimum *z*, PD*_z_*, as a function of *σ* with respect to the optimum *z* estimated for *σ* = 0, can be observed. Similar to what is mentioned above, there is a relative decrease in *z* for a larger *σ*. This decrease is greater at a higher prevalence. For a prevalence of 5%, the maximum decrease in *z* observed is 1.8% when *σ* = 0.614 with respect to the same parameter when *σ* = 0 (as assumed in Dorfman's pooling). On the contrary, for a prevalence of 30%, *z* decreases up to 6.6% when *σ* = 0.614 with respect to when *σ* = 0. This percentage decrease of *z*, PD*_z_*, can be expressed by a power law, PD*_z_* ≈ *bσ*^2^, such that the value of *b* depends on the prevalence. We remark that the value of *R*^2^ in the regression varies from 0.998 to 1.0. Table 3 summarises the function that best fits the respective optimal value of *z* and PD*_z_* for different values of the prevalence.

**Table 3.** Coefficients *a*_0_, *a*_1_, *a*_2_ and *b* to estimate *z* and PD*_z_* as a function of *σ* at different prevalence settings

*x*	*a*_0_	*a*_1_	*a*_2_	*b*
0.05	0.426	−4.55	−0.0206	5
0.1	0.594	−1.45	−0.0459	8.12
0.15	0.718	9.46	−0.0768	8.26
0.2	0.822	−3.36	−0.0896	11.6
0.25	0.912	−5.9	−0.13	15.3
0.3	0.991	−4.42	−0.182	19

### Variations in optimum group size (*n*)

In the initial model (1) (which assumes *σ* = 0), the optimum *n* when *x* = 0.05 is 5, when *x* = 0.1 it is 4, and when *x* is equal to 0.15, 0.2, 0.25 and 0.3 it is 3. In a model that considers heterogeneity, for *x* = 0.05 and *x* = 0.1, the optimum *n* was 5 and 4, respectively, in 149/149 combinations with *σ* from 0.068 to 0.614. For *x* = 0.15, the optimum *n* was 3 in 140/149 combinations, with *σ* from 0.068 to 0.465, while for the nine combinations with a higher *σ*, from 0.49 to 0.614, the optimum *n* was 4. For *x* = 0.2, the optimum *n* was 3 in all 149 combinations. For *x* = 0.25, the optimum *n* was 3 in 148/149 of the combinations between *σ* values of 0.068 and 0.614, with the only exception being the combination of *σ* = 0.525 (the third highest), where the optimum *n* was 4. For *x* = 0.3, the optimum *n* was 3 for the 141 combinations with the lowest *σ*, from 0.068 to 0.49, while of the remaining eight combinations, with *σ* between 0.49 and 0.614, the optimum *n* was 4 for five of them and 3 for the remaining three.

### Standard Dorfman's pooling, Dorfman's pooling by prevalence in age segments and pooling by age segment based on the general prevalence

As mentioned before, three strategies arise when pooling based on prevalence (*x*). The first scenario is standard Dorfman's pooling, where group sizes are calculated by the global prevalence. The second scenario is when samples are separated by age group and pool sizes are calculated based on the prevalence of that specific age segment, which in turn will yield different pool sizes across the segments. The final scenario, and as this model proposes, is to separate by age groups but calculate group sizes in proportion to the global prevalence, which will yield equally sized pools across the segments. Table 4 summarises the different *n* and *z* values predicted by each strategy in the different scenarios.

**Table 4.** Comparison of the pooled strategies mentioned

Age segment	NSW State (AUS)			Hospital Calvo Mackenna (CHL)			Hospital Grant Benavente (CHL)		
	*x* (%)	*n*	*z* (%)	*x* (%)	*n*	*z* (%)	*x* (%)	*n*	*z* (%)
0–19	0.6	13	15.22	22.85	3	87.41	11.71	4	64.24
20–59	1.43	9	23.27	42.5	1	100	16.18	3	74.44
>60	2.26	7	29.07	46.02	1	100	11.93	4	64.84
Global 1	1.52	13, 9, 7	23.66	39.28	3, 1, 1	97.52	14.84	4, 3, 4	71.41
Global 2		9	23.92		1	100		3	71.47
Global 3		9	23.99		1	100		3	71.57

Let us add that Global 1 represents the overall efficiency of Dorfman's pooling separated by age groups. Global 2 represents the overall efficiency of pooling as proposed in this model. Finally, Global 3 represents the efficiency of pooling based on prevalence, without separation into age groups.

To facilitate comparisons between strategies, the prevalence and the *z* values obtained were expressed in their percentage form.

Note that the values for *n* and *z* presented in Table 4 for the strategy proposed in this model (Global 2) are under *σ* values of 0.298, 0.209 and 0.135, respectively. These are the values empirically observed for *σ* in each setting and account for a PD*_z_* of 0 to 0.29%. In contrast, for the other strategies (Global 1 and Global 3), *σ* is assumed to be 0.

## Discussion

As described in the ‘Results’ section, when distributions present higher heterogeneity, better *z* values are observed. A lower value of *z* means greater test savings, since fewer tests are needed to diagnose one subject, and a greater portion of the population can be covered. This inverse relationship between heterogeneity and *z* implies that the limits of the usefulness of the strategy can potentially be stretched to work on an even higher prevalence of positives in a context where the heterogeneity of the population is high and pooling of the samples is separated by age groups.

For most cases, when *γ*_2_ varies from 0.8 to 1.2, the group sizes predicted tend to be equal to the group sizes estimated by Dorfman's pooling. However, it is observed that as *σ* increases, the group size predicted by this model might vary, tending towards greater group sizes at a higher prevalence when *σ* is high. Let us remark that the *z* values achieved are lower in any given context, regardless of the group size estimated, compared to Dorfman's pooling. Although the difference between the *z* predicted might seem minimal when individually comparing *z* values to the efficiency of Dorfman's pooling, this difference might account for significance savings in countries that have implemented mass testing as part of their response strategy. Knowing that the USA is performing between 650 000 and 750 000 tests daily [33], this minimal change in the *z* value might account for significant savings on individual test kits.

However, as shown in Table 4, when looking from the perspective of the three strategies mentioned in this manuscript, the differences in *z* values are rather minimal. Nevertheless, it can be concluded that pooling by age using Dorfman's pooling is the strategy that yields the lowest *z* values, followed by pooling by age groups with equally sized pools throughout the segments and finally standard Dorfman's pooling.

On the contrary, when looking at the applicability of each strategy in the healthcare setting, although applying Dorfman's pooling by age seems to be the most efficient, using this approach will yield different pool sizes across the segments. This, in turn, might signify a greater logistical challenge than separating samples by age and generating equally sized pools throughout the segments, as this model suggests. Furthermore, separating samples by age groups might also signify an additional logistical challenge than not separating the samples at all. Thus, the logistical difficulties of applying each strategy must be individually weighed to implement what is best for each centre.

Nevertheless, when *σ* is taken into account, in settings where there is a high standard deviation in the prevalence of positives within age groups, pooling by these age groups can account for even further savings. When *σ* was at its highest, these savings ranged from 1 extra test for every 14.1 tests in high prevalence settings where the strategy was shown to work, to 1 extra test every 22.2 tests in low prevalence settings.

Finally, it is essential to highlight that although the model predicts local minima at a high prevalence (when *x* ranges from 0.3 to 0.35) for specific settings (such as Australia), this has to be correlated with the *z* value associated with that local minimum. This is because as *z* approaches 1, the overall performance of the strategy becomes similar to individual testing. When *z* equals 1, the same number of tests will be required to cover the same population as individual testing (as was observed for the Hospital Calvo Mackenna), and the strategy is no longer useful. This is important to consider when interpreting the group sizes predicted, as the strategy might be counterproductive, but the model might still show local minima before becoming undefined.

## Final remarks

From the model developed above, it is prudent to conclude that when estimating the optimum number of subjects to include in a pooled sample, separating samples by age groups is a measure that could improve the use of resources compared to the estimation of group size based on the overall prevalence of positives. However, this improvement is minimal when applied on a small scale. Additionally, when a population has high heterogeneity (defined as the standard deviation within confirmed cases among the age segments), the model predicts better performance at a high prevalence than populations with lower heterogeneity under the same prevalence. When compared to standard Dorfman's pooling, separating samples by age segments but governing pool size estimation by the overall prevalence (as proposed in this model), better outcomes are observed. This is mainly objectifiable by the lower *z* values obtained by this method, at any prevalence where the strategy seems to be useful, regardless of the heterogeneity of the population. In this sense, the regression shown in PD*_z_ vs. σ* might serve as a guide to determine the composition of the segments, estimating how the maximum benefit can be obtained and evaluating if this is clinically relevant. This in turn might have an application in establishing screening programmes for children and staff returning to classes, as some schools are starting to reopen. However, for implementing such measures, specific data describing the population under study are key. In this way, age groups can be established based on the possible age ranges that yield the greatest *σ*. From there, the implementation of this strategy could be compared to standard Dorfman's pooling to determine if there is further saving of tests.

Moreover, it is important to emphasise that when facing patients with high clinical suspicion, individual testing should be conducted. Including a highly probable positive case in a pooled sample could potentially mask all of the other negative individuals included in the pooled group, making the use of resources less efficient, as all of the individuals included in the sample will then likely need to be retested individually. Additionally, implementing pool testing in clinical practice might signify a logistical challenge that needs to be addressed to balance the net savings of tests with the extra sample processing times that pooling samples might signify, along with staff availability and training in the matter.

Finally, regarding the prevalence of positives, since the peak of the pandemic has already passed in most countries around the world [21], prevalences over 0.3 are rarely reported. As restrictive measures are slowly lifted and people begin to return to their normal lifestyle, pool testing might serve as a useful monitoring tool to closely monitor the population and quickly detect and isolate new cases that might arise in the upcoming future.

## Data Availability

The data supporting the findings are openly available to the public. From the Australian Government it is available at the NSW webpage, https://data.nsw.gov.au/data/dataset/nsw-covid-19-tests-by-age-range. From the Chilean Government, it is available upon request, subject to the Transparency Law, at https://www.interior.gob.cl/solicitud-de-informacion-ley-de-transparencia/.
